# To live and age as who we really are: Perspectives from older LGBT+ people in Ireland

**DOI:** 10.12688/hrbopenres.12990.2

**Published:** 2020-05-21

**Authors:** Lorna Roe, Miriam Galvin, Laura Booi, Lenisa Brandao, Jorge Leon Salas, Eimear McGlinchey, Dana Walrath

**Affiliations:** 1 Global Brain Health Institute, Trinity College Dublin, the University of Dublin, Dublin, Ireland; 2 Centre for Health Policy and Management, Trinity College Dublin, the University of Dublin, Dublin, Ireland; 3 The Irish Longitudinal Study on Ageing, Trinity College Dublin, the University of Dublin, Dublin, Ireland; 4 Academic Unit of Neurology, School of Medicine, Trinity College Dublin, the University of Dublin, Dublin, Ireland; 5 Discipline of Clinical Medicine, School of Medicine, Trinity College Dublin, the University of Dublin, Dublin, Ireland; 6 Department of Health and Human Communication, Psychology Institute, Federal University of Rio Grande do Sul, Porte Alegre, Brazil; 7 School of Nursing and Midwifery, Trinity College Dublin, the University of Dublin, Dublin, Ireland

**Keywords:** Ageing, diversity, brain health, LGBT+, health inequity

## Abstract

This Open Letter discusses the theme of ‘diversity in brain health’ in research, practice and policy for older LGBT+ people. It is written by a multidisciplinary group of Atlantic Fellows for Equity in Brain Health at the Global Brain Health Institute in Trinity College Dublin (TCD), from a variety of disciplines (health economics, human geography, anthropology, psychology, gerontology) and professions (researcher, clinicians, writers, practicing artists). The group developed a workshop to explore the theme of ‘Diversity and Brain Health’ through the lens of lesbian, gay, bisexual, transgender/transsexual plus (LGBT+).  . Guided by two advisors (Prof Agnes Higgins, TCD; Mr Ciaran McKinney, Age and Opportunity), we invited older LGBT+ people and those interested in the topic of LGBT+ and ageing, healthcare providers, policy makers and interested members of the research community. We partnered with colleagues in the School of Law to include socio-legal perspectives. Following the workshop, Roe and Walrath wrote an opinion editorial, published in the *Irish Times* during the 2019 PRIDE festival, and were subsequently invited by HRB Open Research to provide a more detailed expansion of that work. In this Open Letter we describe the theme of ‘diversity and brain health’ and some of the lessons we learned from listening to the lived experience of older LGBT+ people in Ireland today. We illustrate why it’s important to understand the lived experience of older LGBT+ people and highlight the failure of the State to evaluate the experience of LGBT+ people in policy implementation. We call on researchers, clinicians, service planners and policy makers, to recognize and address diversity as an important way to address health inequities in Ireland.

## Disclaimer

The views expressed in this article are those of the author(s). Publication in HRB Open Research does not imply endorsement by the Health Research Board of Ireland.

## Introduction

Brain health is described as the ability to remember, learn, plan, concentrate and maintain a clear, active mind by being able to draw on the strengths of your brain such as information management, logic, judgement, perspective and wisdom ^1^. A healthy brain functions quickly and automatically. But when problems occur, the results can be devastating. Some of the major types of disorders affecting brain health include: neurogenetic diseases (e.g Huntington’s disease), developmental disorders (e.g. autism spectrum disorder), degenerative diseases of adult life (e.g. Parkinson’s disease and Alzheimer’s disease), metabolic diseases (e.g. Gaucher’s disease), cerebrovascular diseases (e.g. stroke and vascular dementia), trauma (e.g. spinal cord and head injury), convulsive disorders (e.g. epilepsy), infectious diseases (e.g. AIDS dementia), and brain tumors ^2^.

The Global Brain Health Institute (GBHI) was established to promote equity in brain health, specifically to address the risk and impact of dementia globally. Dementia - an umbrella term for a variety of diseases of the brain which cause cognitive decline and loss of function - is incurable, but, a range of non/pharmacological therapies exist to manage the condition ^3^. Dementia is determined by a complex range of risk factors including genetic (e.g. ApoE e4 gene), lifestyle (e.g. smoking, exercise), health (e.g. cardiovascular risks, depression, hearing loss), social (e.g. loneliness, isolation) and environment (e.g. pollutants) ^3^. It’s been estimated that 1 in 3 dementia cases could potentially be prevented by addressing risk factors ^4^.

## Diversity and brain health

Differential exposure to social, economic, and environmental risks factors between individuals lead to health inequities, which are defined as differences in health which are systematic, socially produced (and therefore modifiable), and unfair ^5^. Such inequities are not occasional or random, rather they are significant, frequent, or persistent associations ^6^. For example, in Ireland an additional 10.2 infant deaths per 1,000 live births are found in the Irish Traveller community (an Irish ethnic minority group) compared to the general population ^7^; 30 year differences in mortality are found in adults who are homeless in Dublin compared to the general population ^8^, and a 2.80 beats per minute higher resting heart rate (a risk factor for cardiovascular disease in older adults) are found in older adults in the lowest compared to the highest income quintile ^9^. Systematic differences in brain health have also been found. For example, a higher prevalence of cognitive impairment has been found among adults who are homeless in California compared to the general population ^10^.

In seeking to address health inequities, epidemiologist Sir Michael Marmot, Chair of the Commission on the Social Determinants of Health (SDH), urged us to address the ‘ *causes of causes’* by tackling the conditions of life for people as they are born, live, work and age ^11^.

**Figure 1.**
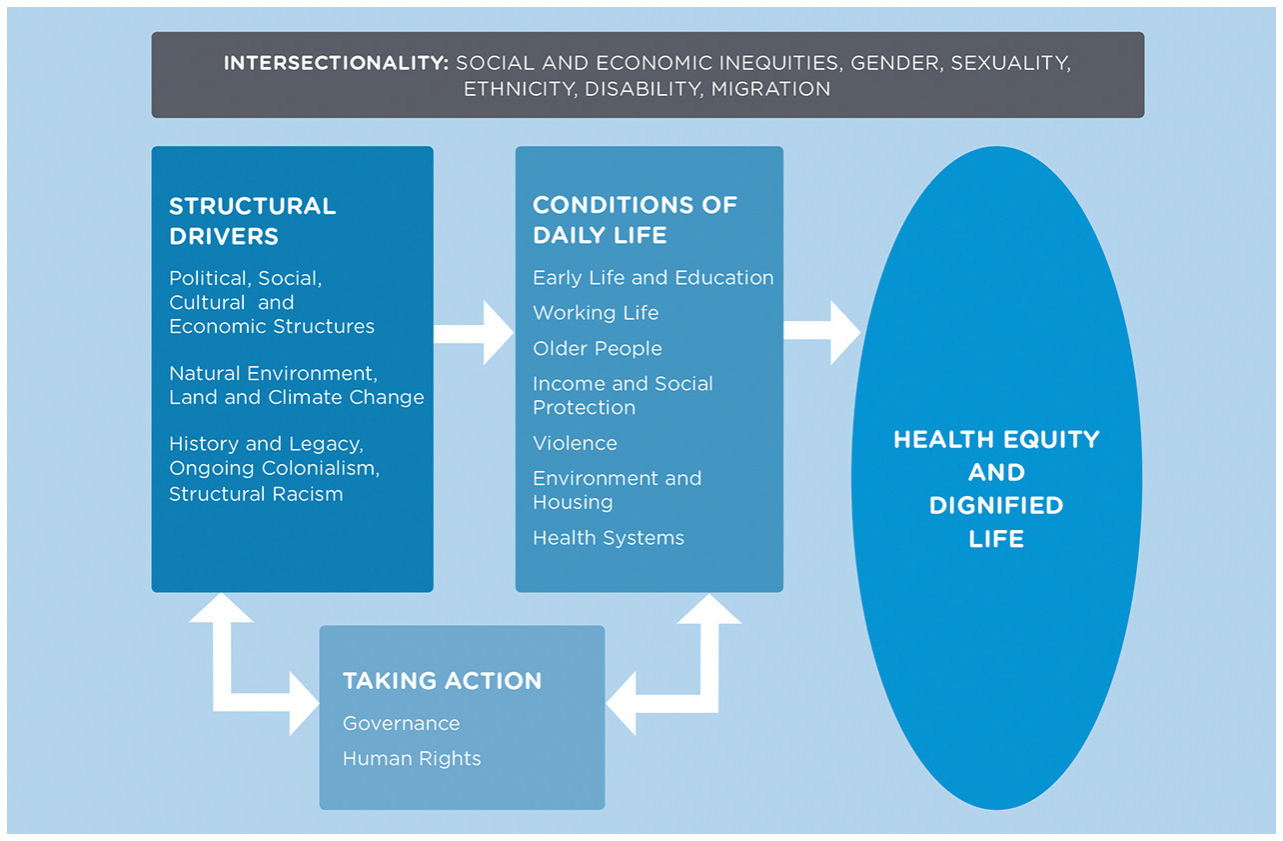
Conceptual Framework of the Pan American Health Organization (PAHO) Commission on Equity and Health Inequalities in the Americas report (Marmot 2018). Figure reprinted from The Lancet, 392(10161) Marmot M, Just societies, health equity, and dignified lives: the PAHO Equity Commission., Pages No. 2247-2250, Copyright (2018), with permission from Elsevier.

In 2018, Sir Marmot presiding over the Pan American Health Organization’s (PAHO) Commission on Equity and Health Inequalities in the Americas report, expanded the SDH framework to include structural drivers (e.g. institutional racism) and the intersection of various drivers as factors which adversely increase the experience of the social determinants of health, see Figure 1 ^12^.

**Figure 2.**
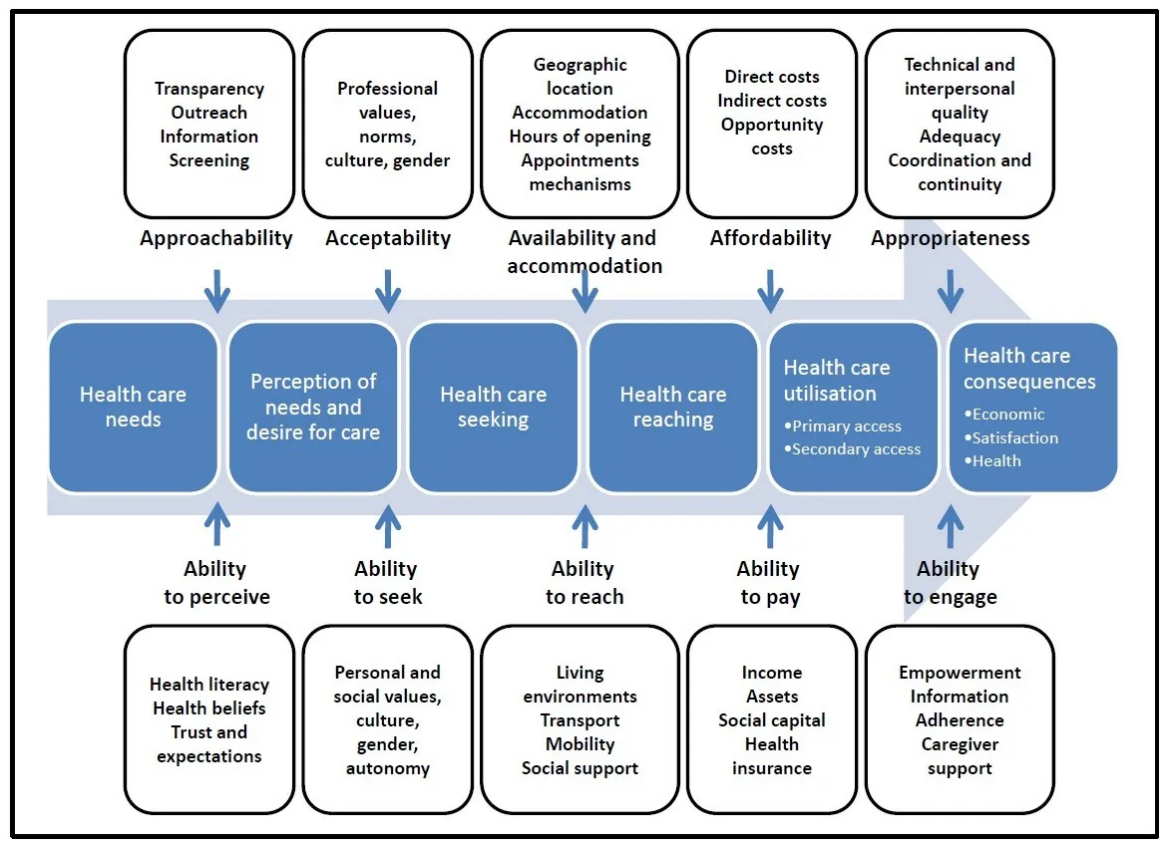
A conceptual framework of access to health care (Levesque *et al.* 2013). Figure reproduced from (Levenesque *et al.* 2013) under the terms of the Creative Commons Attribution 2.0 Generic License (CC BY 2.0) which permits unrestricted use, distribution, and reproduction in any medium, provided the original work is properly cited.

*Structural drivers,* or structural violence, is a term coined by anthropologists, which describes how oppressive social structures or institutions can result in death, injury, illness, subjugation, stigmatization, and even psychological terror for specific social groups or populations ^13^. Structural factors are borne out as determinants in the brain health literature, such as level of education attainment, which is impacted by many factors, including the quality of education. For example, in the United States, schools in the South were racially segregated up to 1954, with schools for African American students on average receiving fewer resources (e.g. shorter school term length, higher pupil-teacher ratio) than schools for white students ^14^. Attendance at schools in southern states was subsequently found to be associated with years of completed education and late-life cognitive decline ^14^. Structural factors are also important in the context of healthcare systems which can also influence health outcomes. For example, long waiting times in the Emergency Department can be a barrier for individuals with addiction issues or attention deficit hyperactivity disorder, common within the homeless population ^15^. Access to health care is defined as the opportunity to reach and obtain appropriate health care services in situations of perceived need for care ^16^. Components of access include ‘approachability’ (people can identify their health needs and the services needed to meet these needs) ‘acceptability’ (people accept the socio-cultural aspects of the service); ‘availability’ (an adequate supply of services relative to needs); ‘accommodation’ (health services can be reached both physically and in a timely manner); ‘affordability’ (the in/direct cost implications to the person in relation to need); and ‘appropriateness’ (fit between services and person’s needs), see Figure 2 ^16^.

*Intersectionality* refers to the complexities of how people experience disadvantage based on a broad array of social group memberships (e.g. race, class, religion, sexual orientation, ability and gender) ^17^. Cultural and political processes produce each of these aspects of identity. Each person has a social location where their identities overlap, which determines their existence in the social and political world, their relationship to others and to dominant cultures, and the kinds of power and privilege they have access to and can exercise ^17^. Across the world, identity is used to influence the distribution of power and privilege and both sub/conscious oppressive actions maintain the status quo. Oppressive actions are expressed by individuals (e.g. attitudes and behaviours), institutions (e.g. policies, practices and norms), and society/culture (e.g. values, beliefs and customs). These actions do not ‘just happen’, rather they are reproduced in a process of normalization and reinforced in a cycle of ‘business as usual’. It’s possible to interrupt oppressive cycles by calling into question the truth of what is learned about the power relationships among different social groups and our own social position ^17^.

## Diversity and brain health: Perspectives from older LGBT+ people

In this context, the workshop was developed by a group of Fellows who were aware that lesbian, gay, bisexual, transgender/transsexual plus (LGBT+) people were and are not always accepted both within and across countries. In 2019 being LGBT+ was illegal in 68 countries worldwide and punishable by death in 12 countries ^18^. Furthermore, researchers have reported experiencing barriers to publication with an LGBT focus without severe risk of harm to themselves, their colleagues, and their families ^19^. We were also aware these identities can go unseen in healthcare settings. Through our clinical rotations (as part of the Fellowship programme), we were cognizant that for some people, changes in cognition associated with dementia (affecting an estimated 55,000 older Irish adults), could result in a disclosure of their true gender or sexuality for the first time. We agreed that the older LGBT+ community would be a valuable lens through which to look at diversity and to learn about how diverse identities impact the experience of ageing, interactions with healthcare services, and the ability to ‘age in place’. Ireland provided an interesting case example having transitioned recently and rapidly from a State in which homosexuality was a crime, to one which welcomed marriage equality and gender recognition legislation. Together with two advisors ^1^ and with partners in the School of Law at TCD ^2^, we developed a workshop held on June 4 ^th^ at TCD, to examine diversity and brain health through the lens of the LGBT+ community ^3^. Following the workshop, Roe and Walrath wrote an opinion editorial, published in the *Irish Times* during the 2019 PRIDE festival ^20^ and were subsequently invited by HRB Open Research to provide a more detailed expansion of that work.

Below, we outline 10 things we learned about diversity and brain health, by listening to the lived experience of older LGBT+ people and their advocates.

1. **The ‘LGBT+’ experience:** It’s easy to think there is a common ‘LGBT+ experience’, when we view this identity through a heteronormative lens. In reality, variation exists between different sexualities and gender identities, which intersect with other factors, such as social class. For example, some attendees spoke about the psychological stress of needing to hide their identities in their workplaces. For some, their identity would have led to expulsion due to the explicit rules of the organization, while others felt the stigma would have serious repercussions for their jobs or businesses. By contrast, other participants spoke with pride about being part of a community which supported one another and a social movement which brought about positive change. It became clear that LGBT+ specific protective factors (e.g. increased resilience) and risk factors (e.g. internalized stigma or disenfranchised grief ^21^) to general health and brain health, were not experienced universally within this community. Therefore; while we need to examine the experiences and outcomes of LGBT+ people as a social group within the general population, it is important to examine the within-group variation with respect to the nature and distribution of protective and risk factors.

2. **Dissonant identities:** Some LGBT+ people experienced dissonance between their LGBT+ identity and other identities. For example, an attendee described coming under pressure to ‘drop’ her LGBT+ identity to be accepted as grandmother to her new grandchild. Sociologists describe this as a ‘psychological colonization’ where oppressed group members knowingly, but not necessarily voluntarily, go along with their own mistreatment to survive or to maintain some status, livelihood, or other benefit ^17^. Her narrative of hidden identity, resonates with a story recently published in the *Irish Times*, of a closeted gay man who internalized stigma, and kept secret from his family his (requited) love for another man until his death ^22^. 

3. **The attitudes of healthcare providers:** We learned how healthcare services can be ill prepared to accommodate LGBT+ people. For example, a transgender woman described receiving a single room in hospital, although she didn’t have private health insurance, something she felt was because health care staff felt she did not fit into the ‘male’ or ‘female’ wards. This could be seen as an example of a subconscious (i.e. implicit) bias if organisations such as hospitals fail to develop policies to guide staff. For others, issues of discrimination at the societal level and lack of adequate training for health care professionals may lead to negative experiences which colour future interactions with healthcare practitioners. Attendees noted a particular strain regarding homecare workers whose personal beliefs interfered with providing respectful compassionate care to members of the LGBT+ community. This could be seen as an example of conscious (i.e. explicit) bias, where a staff member knowingly commented in a stigmatising or derogatory way to a service user. Workshop attendees also called for the presence of LGBT+ individuals as well as allies at every level of health services.

4. **Source of social support and caregiving**: For some attendees a ‘chosen family’ comprising emotional and social ties, is as important as a biological or legal family. This was especially the case before the legal recognition of same sex marriage, as networks of peers, friends and non-relatives are a major source of support and, importantly, know the person’s care and the end of life preferences. These individuals or chosen families need to be recognized and included by clinicians and care providers in decision-making discussions. This could be done for example, by asking in a clinical encounter “Is there anyone important to you that you would like involved now?” instead of assuming that the biological family is the only or most appropriate source.

5. **History and healthy ageing:** For some workshop attendees, previous negative experiences in society, or simply their preference to be with people from the LGBT+ community, makes services such as day centres or even care homes which have a predominantly heteronormative culture, an uncomfortable space. This is important, as by failing to appreciate the effects of historical stigma and discrimination, we are in danger of “seeing the puddles, but not the rainstorms and certainly not the gathering thunderclouds” ^13^, and running the risk of creating barriers to care which will perpetuate health inequities ^23^. By this we mean failing to address how older LGBT+ people may not feel accepted in these spaces due to fear or stigma.

6. **Access to homecare:** In Ireland, homecare is wedded to the nuclear family, particularly the role of women who make up the majority of our informal or family carers. The State’s role in homecare is defined by the principle of subsidiarity in care and social matters - family first and State second - a legacy of our socially conservative history ^24^. Consequently, State provided homecare often only supplements informal care, offering enough hours to support an older adult, only if informal care can cover the remainder hours ^24, 25^. All this means those with non-traditional family arrangements, typical within the LGBT+ community, are less likely to receive homecare and the only option becomes costly residential care.

7. **Ageing and health policy:** To address health inequities and social exclusion, strategic commitments must be supported by mechanisms such as target setting, monitoring and evaluation underpinned by adequate data collection to measure progress. However, in Ireland, though the Government identifies older LGBT+ people as a group at risk of social exclusion, it has yet to monitor their experiences or develop bespoke policy solutions which support their inclusion. For example, while LGBT+ people are identified as a group vulnerable to social exclusion in the National Positive Ageing Strategy ^26^, their experiences are not captured by the national indicators to monitor the strategies’ implementation ^27, 28^.

8. **Social inclusion and LGBT+ communities:** We learned how activities designed for geographically-based communities fail to address the needs of dispersed communities without spatial boundaries. For example, geographically-based initiatives such as Men’s Sheds have been enormously successful in supporting healthy ageing among men. However, older lesbians across the country who historically met-up to support each other, are now at risk of isolation and loneliness in old age as they cannot secure physical spaces in urban areas because of the competition for those resources.

9. **Harnessing the strength of diverse social groups:** The successful fight for social recognition of diverse gender and sexual identities highlights the skills, knowledge, and vocabulary that this community can bring to the identity politics of ageing. This community knows how to support one another through the formation of community groups such as OWLS, GOLD, Running Amach, and Outhouse, how to fight for services that do not exist, how to coin terms for concepts society only knows subconsciously.

10. **Being inclusive:** Attendees remarked the needs of older LGBT+ people will change over time. The experience of being LGBT+ people today is different to what it was historically, with shifts in social attitudes, legal rights and the language. Thus, the solutions to create an inclusive society are not neat interventions. Rather inclusivity needs to be a value, and ‘being inclusive’ recognized as a process which focuses on what is meaningful to people, what facilitates people to be themselves in the world, to age as they are and to leave it as who they are. For LGBT+ people this means being able to maintain their identity through ageing and end of life. Learning how to be inclusive to LGBT+ people will teach us how to be inclusive to all forms of diversity, including those living with diseases of the brain ^29^.

## Conclusion

We learned the older LGBT+ community experience healthcare services and ageing in place in different ways. This community comprises several sub-communities which play an important role in the lives of its members by providing social outlet and support; containing a rich reservoir of history and identity; and being highly resourced in advocacy and self-expression. As older LGBT+ people age, they are finding ways to harness the opportunities which ageing brings, while learning to adapt to their changing personal circumstances. Some of the challenges faced by this community can include negative interactions with healthcare workers and the design of societal structures and policies which fail to address their specific needs. These factors can negatively affect older LGBT+ people in terms of a heightened risk of loneliness and discrimination in old age, their ability to access inclusive person-centred care where they feel safe and accepted, and their ability to continue to fully participate in society while maintaining their identity. 

The issues raised by the workshop participants challenge our societal responsiveness on a number of levels. At the level of the healthcare system, issues such as these speak to the *appropriateness* of healthcare services and delivery, rather than simply to the *availability* of services, often the most commonly spoken about barrier to accessing care in the Irish context. At the level of social policies, these issues speak to the need for the design of policies for the population, not blind to the specific needs of sub-groups, such as older LGBT+ people. At the individual level, these issues speak to the lack of skills and language which would make everyday interactions more accommodating, inclusive and welcoming for older LGBT+ people. 

In conclusion, by learning how to become more inclusive of the LGBT+ community and their needs, we learn the skill of being an accepting and inclusive society to all forms of diversity. And if we can be respectful of differences of whatever kind and develop inclusive services and policies, we can address structural and intersectional factors that impact on healthy ageing. We call on researchers, clinicians, service planners and policy makers to recognize the importance of knowing their own ‘social location’ and how it might blind them to the needs and experiences of diverse groups in their work. 

## Recommendations

We call specifically for the research, policy and health care community to:

➢   Collect data to identify LGBT+ people in quantitative studies on ageing and consider how this identity intersects with other factors to create health inequities in old age. Undertake qualitative research to understand the ways in which discrimination and stigma affects older LGBT+ people at individual, institutional, societal and cultural levels.

➢   Provide diversity-awareness training programmes ^4^ which gives healthcare professionals the language and skills to identify and support older LGBT+ people in clinical practice; including the identification and inclusion of an individuals’ ‘chosen family’ in medical and social decisions.

➢   Develop policies which harness the existing strengths and skills (i.e. intrinsic capacity) of diverse groups, which are socially rather than geographically defined, in the promotion of healthy ageing.

➢   Evaluate strategies designed to improve access to healthcare and to address social exclusion by monitoring the experiences and outcomes of diverse social groups, such as older LGBT+ people. 

➢   Consider how best to support older LGBT+ people with homecare where no informal care is available.

➢   Recognise that 'inclusivity' is not a standalone intervention, but an ongoing process, allowing people to live, age and die as who they wish to be.

## Data availability

### Underlying data

No data are associated with this article

## Notes

^1^ *Advisors*: Prof Agnes Higgins, Professor in Mental Health, School of Nursing & Midwifery at TCD and the author of Visible Lives report (Higgins *et al.*, 2011), Ciaran McKinney, Engage Programme Manager (Promoting active citizenship and lifelong learning) at Age & Opportunity, formerly Director of Gay HIV Strategies at Gay and Lesbian Equality Network.

^2^ *Partners*: From the School of Law in TCD, Prof Mark Bell and Prof Mary Rogan contributed to workshop planning, while Prof Ivana Bacik presented during the workshop.

^3^ 85 people registered for the workshop on June 4 ^th^ 2019 from organisations representing the LGBT+ community e.g Gay and Lesbian Equality Network, Older Women Lesbians and Transgender Equality Network, Running Amach, and LGBT Ireland. Delegates also came from the Department of Health, charities representing older adults and academia. Research Ast. Prof Roe and Dr Walrath led the workshop with contributions invited from: Senator Davis Norris, Marianne McGiffin (Older Wiser Lesbians), Claire Farrell (Transgender Equality Network Ireland), Ciaran McKinney (Age and Opportunity), Prof Agnes Higgins (School of Nursing, TCD), Prof Victor Valcour (GBHI- UCSF), Prof Brian Lawlor (GBHI- TCD), Prof Mark Bell (School of Law, TCD), Prof Ivana Bacik (School of Law, TCD), Amanda Dunsmore (Limerick School of Art & Design, LIT ), Karl Duff (Department of Health, Ireland). During the workshop, Dr Laura Booi (GBHI-TCD) and Ast. Prof Eimear McGlinchey (GBHI-TCD) took written feedback from attendees on post-its and Prof Mary Rogan (School of Law, TCD) and Prof Miriam Galvin (GBHI-TCD) took notes on the workshop themes and provided a synthesizing wrap up.

^4^ (Note: Since the workshop, LGBT Ireland have been awarded funding from the Slaintecare Integration Fund Project for their training programme for health and social care professionals working in older age services.)
