# Supplementary material for: To live and age as who we really are: Perspectives from older LGBT+ people in Ireland
Source: HRB Open Res. 2020 May 21;3:6. Originally published 2020 Feb 12. [Version 2] doi: 10.12688/hrbopenres.12990.2 (PMC7141166; doi:10.12688/hrbopenres.12990.2)
Supplement: Peer review history

# Peer review: Reviewer response for version 2

**DOI:** 10.21956/hrbopenres.14162.r27431

**Published:** 2020-06-03

**Authors:** Jessica Marsack

**Affiliations:** 1 Department of Health Behavior and Biological Sciences, School of Nursing, University of Michigan, Ann Arbor, MI, USA

No additional comments.

Does the article adequately reference differing views and opinions?

Partly

Are all factual statements correct, and are statements and arguments made adequately supported by citations?

Yes

Is the Open Letter written in accessible language?

Yes

Where applicable, are recommendations and next steps explained clearly for others to follow?

Yes

Is the rationale for the Open Letter provided in sufficient detail?

Yes

Reviewer Expertise:

Health disparities, Sexual and gender minority health, Internal medicine, Kidney failure & dialysis, End of life care.

I confirm that I have read this submission and believe that I have an appropriate level of expertise to confirm that it is of an acceptable scientific standard.

# Peer review: Reviewer response for version 2

**DOI:** 10.21956/hrbopenres.14162.r27432

**Published:** 2020-05-28

**Authors:** Caroline Dorsen

**Affiliations:** 1 Rory Meyers College of Nursing, New York University, New York, NY, USA

I have no further comments. Thank you again for the opportunity to review this manuscript. Looking forward to future collaborations.

Does the article adequately reference differing views and opinions?

Yes

Are all factual statements correct, and are statements and arguments made adequately supported by citations?

Partly

Is the Open Letter written in accessible language?

Partly

Where applicable, are recommendations and next steps explained clearly for others to follow?

Yes

Is the rationale for the Open Letter provided in sufficient detail?

Yes

Reviewer Expertise:

LGBTQ health disparities with a focus on stigmatizing and affirming care from healthcare providers and how they impact sexual risk and substance use behaviors among sexual and gender minority persons.

I confirm that I have read this submission and believe that I have an appropriate level of expertise to confirm that it is of an acceptable scientific standard.

# Peer review: Reviewer response for version 2

**DOI:** 10.21956/hrbopenres.14162.r27433

**Published:** 2020-05-21

**Authors:** Gloria Gutman

**Affiliations:** 1 Gerontology Department, Simon Fraser University, Vancouver, Canada

No further comments.

Does the article adequately reference differing views and opinions?

Yes

Are all factual statements correct, and are statements and arguments made adequately supported by citations?

Yes

Is the Open Letter written in accessible language?

Partly

Where applicable, are recommendations and next steps explained clearly for others to follow?

Yes

Is the rationale for the Open Letter provided in sufficient detail?

Partly

Reviewer Expertise:

Health promotion and aging, LGBT aging, gerontechnology, aging and the built environment, disasters and aging, elder abuse prevention and mitigation.

I confirm that I have read this submission and believe that I have an appropriate level of expertise to confirm that it is of an acceptable scientific standard.

# Peer review: Reviewer response for version 1

**DOI:** 10.21956/hrbopenres.14076.r27175

**Published:** 2020-04-07

**Authors:** Gloria Gutman

**Affiliations:** 1 Gerontology Department, Simon Fraser University, Vancouver, Canada

I agree with reviewer 1 that if this open letter is directed to an international readership it would be helpful if terms known mainly to the Irish population were explained. I needed to go to Google to determine that Travellers were an ethic minority and to learn that “rough sleepers” were what I believe elsewhere are referred to as the homeless population. My main recommendation however is that consideration be given to modifying the title, which is a bit misleading. The main argument of the letter, which is an important one, is the need for inclusivity in research, practice and policy related to brain health. We need the voices of marginalized groups such as the LGBT population to be heard, and their lived experiences to be recognized but also we need hard data i.e. numbers with respect to incidence and prevalence of neurodegenerative diseases such as dementia affecting this population – which is what at first I thought I might find in the article. We also need to recognize the “diversity within diversity” reflected in learnings #1 and #10. In the case of research, this requires data collection and analysis to explore and to “slice and dice” the LGBT population. Although not stated in the letter, to date the preponderance of research is on gay men. Services and service providers need to be reminded, as the letter suggests, that this is not a homogeneous group. In general, the learnings part of the letter are excellent and concordant with research from elsewhere. They could be tied back a bit more to the theme and to dementia and other of the conditions the research group specializes in but otherwise, the authors are to be congratulated for conducting the workshop and sharing their findings via the open letter.

Does the article adequately reference differing views and opinions?

Yes

Are all factual statements correct, and are statements and arguments made adequately supported by citations?

Yes

Is the Open Letter written in accessible language?

Partly

Where applicable, are recommendations and next steps explained clearly for others to follow?

Yes

Is the rationale for the Open Letter provided in sufficient detail?

Partly

Reviewer Expertise:

Health promotion and aging, LGBT aging, gerontechnology, aging and the built environment, disasters and aging, elder abuse prevention and mitigation.

I confirm that I have read this submission and believe that I have an appropriate level of expertise to confirm that it is of an acceptable scientific standard.

# Response

**Published:** 2020-05-15

**Authors:** Lorna Roe

**Affiliations:** Trinity College Dublin, Ireland

Thank you for your time in reviewing our article. We recognize that the term ‘travellers’ needs to be described for an international audience and have amended the text with reference to the meaning of the term in the Irish context. We are grateful for your suggestion to change the title to reflect the article content more clearly, the revised title is ‘ *To live and age as who we really are: Perspectives from older LGBT+ people in Ireland’*.

It was beyond the scope of the workshop to collect any self-reported or objective measures of brain health from those attending. However, we intend to pursue this in our future work on diversity and brain health. Finally, we added a section examining the higher level learnings from this workshop in relation to the theme of brain health and diversity. And we intend to continue to contribute to this area, given the gaps in knowledge you have outlined.

# Peer review: Reviewer response for version 1

**DOI:** 10.21956/hrbopenres.14076.r27226

**Published:** 2020-03-31

**Authors:** Caroline Dorsen

**Affiliations:** 1 Rory Meyers College of Nursing, New York University, New York, NY, USA

Thank you for the opportunity to review your paper 'To live and age as who we really are". This is a very timely and important topic as we face the global ageing of the population, with relatively little understanding of the unique culture and special health and social needs of older LGBTQ+ persons. I hope my comments below are helpful as you prepare your manuscript. 

 Please note that as this is not a research report, my review does not follow the usual protocol. 

1. Overall, I liked the paper content and structure very much, and found it was informative and written in a useful way for both health and social care providers. Well done. Most of my revision suggestions are small.
2. I found the writing clear, although there are a few places that the authors use language that is perhaps not understood by non-Irish readers. For example, 'rough sleepers" is not a term we use in the US. I raise this issue to make sure that you are understood by the maximum number of readers, especially because you give examples from the US, specifically making the paper of interest to North Americans.
3. Similarly, you use a number of terms in the paper that would not be acceptable to readers from the US. The clearest example of this is describing LGBTQ+ persons as 'non-normative.' This is reflective of a very heteronormative view of sexual orientation and edgier identity and would be quite offensive to some LGBTQ+ people and allies.
4. There is a sentence fragment 'While a lesbian...'
5. I would like to see you differentiate between subconscious bias (or implicit) and conscious (or explicit) bias in the attitudes section. This is essential for understanding the nuances of the barriers to access to and utilization of care among LGBTQ+ persons. 
6. I am glad that you mentioned the historical moment and subsequent shifts in social and legal norms. I think it is important to say something here about the fact that we experience significant controversies around the globe about LGBTQ rights, and that patients and families may have had a huge variation of experiences previously, and sense of safety currently. There are over 70 countries in the world where it is still illegal to be LGBTQ, in about 15 of them LGBTQ is a crime punishable by death.
7. Excellent recommendations for the future.

Does the article adequately reference differing views and opinions?

Yes

Are all factual statements correct, and are statements and arguments made adequately supported by citations?

Partly

Is the Open Letter written in accessible language?

Partly

Where applicable, are recommendations and next steps explained clearly for others to follow?

Yes

Is the rationale for the Open Letter provided in sufficient detail?

Yes

Reviewer Expertise:

LGBTQ health disparities with a focus on stigmatizing and affirming care from healthcare providers and how they impact sexual risk and substance use behaviors among sexual and gender minority persons.

I confirm that I have read this submission and believe that I have an appropriate level of expertise to confirm that it is of an acceptable scientific standard, however I have significant reservations, as outlined above.

# Response

**Published:** 2020-05-15

**Authors:** Lorna Roe

**Affiliations:** Trinity College Dublin, Ireland

Thank you for your time in reviewing our article. We have addressed the issues you have raised and amended the original text. The term ‘rough sleepers’ is changed to ‘adults who are homeless’; we have deleted ‘non-normative’. We have addressed the sentence fragment identified in the text. We illustrated where there may be examples of subconscious and conscious bias in some of the issues described by workshop participants. However this requires further work exploring the attitudes and behaviours of people towards older LGBT+ people to fully understand sub/conscious bias. We recognise that being LGBT+ is unsafe in many countries around the world and have included additional text to explicitly refer to this issue.

The authors appreciate your contribution and feel that the article is stronger because of the points that you raised and the amendments in the text.

# Peer review: Reviewer response for version 1

**DOI:** 10.21956/hrbopenres.14076.r27229

**Published:** 2020-03-16

**Authors:** Jessica Marsack

**Affiliations:** 1 Department of Health Behavior and Biological Sciences, School of Nursing, University of Michigan, Ann Arbor, MI, USA

This open letter is a wonderful expanded discussion of an opinion piece published in the Irish Times. The open letter as a whole discusses the authors' "lessons learned" from a workshop held around the aging LGBT+ community. The rationale for this article is clearly stated with good background information and relevant citations, so the factual statements made in the article are covered. However, the criteria "does the article adequately reference differing views and opinions?" is only partly covered. While the article does mention some opposing opinions, e.g. under #3 where the attitudes of healthcare providers are described, the majority of the article is about one viewpoint. Obviously the article is going to focus on this viewpoint because it is discussing a workshop around a singular viewpoint, but it may be worthwhile to lend some citations to the opposition rather than just case evidence. My only comment about language being "accessible" is really in relation to description of transgender individuals - for example, the use of the phrase "trans-woman" in #3. Wording such as "transfeminine person" might be more inclusive, or "transgender woman" more formal. The next steps and recommendations are clearly laid out, reasonable, and described in some detail.

Does the article adequately reference differing views and opinions?

Partly

Are all factual statements correct, and are statements and arguments made adequately supported by citations?

Yes

Is the Open Letter written in accessible language?

Yes

Where applicable, are recommendations and next steps explained clearly for others to follow?

Yes

Is the rationale for the Open Letter provided in sufficient detail?

Yes

Reviewer Expertise:

Health disparities, Sexual and gender minority health, Internal medicine, Kidney failure & dialysis, End of life care.

I confirm that I have read this submission and believe that I have an appropriate level of expertise to confirm that it is of an acceptable scientific standard.

# Response

**Published:** 2020-05-15

**Authors:** Lorna Roe

**Affiliations:** Trinity College Dublin, Ireland

Thank you for your time in reviewing our article. We reviewed some of the language used to describe LGBT+ people in keeping with international norms and your suggestion regarding the accessibility of the term ‘trans-woman’. This has been changed to ‘transgender woman’ in the text.

In relation to including opposing perspectives and arguments in this article, we feel that the article as currently constructed reports on the workshop proceedings and views expressed therein. We agree that alternative perspectives are important in order to get a more comprehensive view of LGBT communities and brain health. This was beyond the scope of this particular piece of work, but we aim to conduct a systematic literature review in our future work in this topic area which will capture divergent views and alternative perspectives.
